# Health Benefits and Pharmacological Properties of Stigmasterol

**DOI:** 10.3390/antiox11101912

**Published:** 2022-09-27

**Authors:** Saad Bakrim, Nesrine Benkhaira, Ilhame Bourais, Taoufiq Benali, Learn-Han Lee, Nasreddine El Omari, Ryan A. Sheikh, Khang Wen Goh, Long Chiau Ming, Abdelhakim Bouyahya

**Affiliations:** 1 Molecular Engineering, Biotechnologies and Innovation Team, Geo-Bio-Environment Engineering and Innovation Laboratory, Polydisciplinary Faculty of Taroudant, Ibn Zohr University, Agadir 80000, Morocco; 2 Laboratory of Microbial Biotechnology and Bioactive Molecules, Department of Biology, Faculty of Sciences and Techniques, University Sidi Mohamed Ben Abdellah, Fez 1975, Morocco; 3 Laboratory of Human Pathologies Biology, Department of Biology, Faculty of Sciences, Mohammed V University in Rabat, Rabat 10106, Morocco; 4 Environment and Health Team, Polydisciplinary Faculty of Safi, Cadi Ayyad University, Sidi Bouzid B.P. 4162, Morocco; 5 Novel Bacteria and Drug Discovery Research Group (NBDD), Microbiome and Bioresource Research Strength (MBRS), Jeffrey Cheah School of Medicine and Health Sciences, Monash University Malaysia, Bandar Sunway 47500, Malaysia; 6 Laboratory of Histology, Embryology, and Cytogenetic, Faculty of Medicine and Pharmacy, Mohammed V University in Rabat, Rabat 10100, Morocco; 7 Biochemistry Department, Faculty of Science, King Abdulaziz University, Jeddah 21589, Saudi Arabia; 8 Faculty of Data Science and Information Technology, INTI International University, Nilai 71800, Malaysia; 9 PAP Rashidah Sa’adatul Bolkiah Institute of Health Sciences, Universiti Brunei Darussalam, Gadong BE1410, Brunei

**Keywords:** stigmasterol, pharmacological activities, anticancer, anti-inflammatory, immunomodulatory

## Abstract

Stigmasterol is an unsaturated phytosterol belonging to the class of tetracyclic triterpenes. It is one of the most common plant sterols, found in a variety of natural sources, including vegetable fats or oils from many plants. Currently, stigmasterol has been examined via in vitro and in vivo assays and molecular docking for its various biological activities on different metabolic disorders. The findings indicate potent pharmacological effects such as anticancer, anti-osteoarthritis, anti-inflammatory, anti-diabetic, immunomodulatory, antiparasitic, antifungal, antibacterial, antioxidant, and neuroprotective properties. Indeed, stigmasterol from plants and algae is a promising molecule in the development of drugs for cancer therapy by triggering intracellular signaling pathways in numerous cancers. It acts on the Akt/mTOR and JAK/STAT pathways in ovarian and gastric cancers. In addition, stigmasterol markedly disrupted angiogenesis in human cholangiocarcinoma by tumor necrosis factor-α (TNF-α) and vascular endothelial growth factor receptor-2 (VEGFR-2) signaling down-regulation. The association of stigmasterol and sorafenib promoted caspase-3 activity and down-regulated levels of the anti-apoptotic protein Bcl-2 in breast cancer. Antioxidant activities ensuring lipid peroxidation and DNA damage lowering conferred to stigmasterol chemoprotective activities in skin cancer. Reactive oxygen species (ROS) regulation also contributes to the neuroprotective effects of stigmasterol, as well as dopamine depletion and acetylcholinesterase inhibition. The anti-inflammatory properties of phytosterols involve the production of anti-inflammatory cytokines, the decrease in inflammatory mediator release, and the inhibition of inducible nitric oxide synthase (iNOS) and cyclooxygenase-2 (COX-2). Stigmasterol exerts anti-diabetic effects by reducing fasting glucose, serum insulin levels, and oral glucose tolerance. Other findings showed the antiparasitic activities of this molecule against certain strains of parasites such as *Trypanosoma congolense* (in vivo) and on promastigotes and amastigotes of the *Leishmania major* (in vitro). Some stigmasterol-rich plants were able to inhibit *Candida albicans*, *virusei*, and *tropicalis* at low doses. Accordingly, this review outlines key insights into the pharmacological abilities of stigmasterol and the specific mechanisms of action underlying some of these effects. Additionally, further investigation regarding pharmacodynamics, pharmacokinetics, and toxicology is recommended.

## 1. Introduction

The development of drugs against human pathologies dates back a long time, with the traditional use of natural resources such as medicinal plants. Indeed, with the advancement of molecular biology, anatomopathology and system biology, complex pathologies such as diabetes, cancer, and chronic inflammation are beginning to be deciphered [1]. Furthermore, the search for molecules with pharmacological properties against these pathologies has become increasingly molecular. Moreover, certain molecular mechanisms involved in these pathologies seem to be similar for the different pathologies, which makes the actions of the bioactive substances diverse and multiply; a single molecule can have several pharmacological effects when it acts on subcellular, cellular, and molecular targets involved in these pathologies [2,3,4]. Moreover, certain subcellular pharmacodynamic actions of the same molecule can have amplified and divergent therapeutic effects, which justifies the different pharmacological properties of certain bioactive molecules.

**Figure 1:**
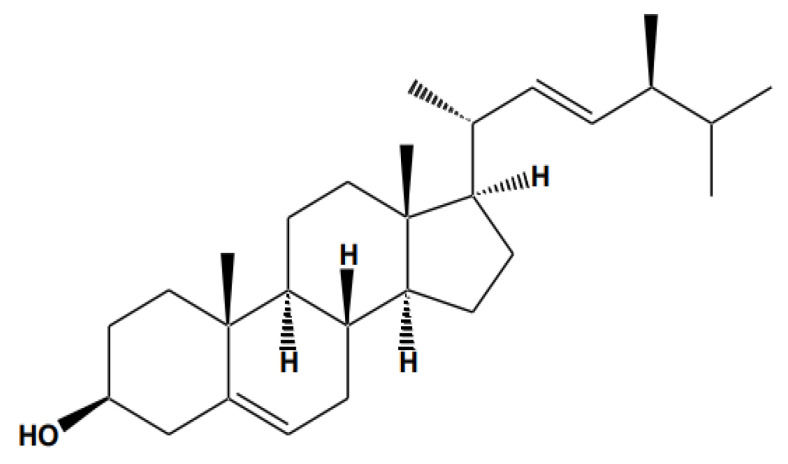
Structural formulae of stigmasterol.

Stigmasterol (C_29_H_48_O, stigmasta-5,22-dien-3-ol), also known as wulzen anti-stiffness factor or stigmasterin, is a phytosterol, i.e., a steroid belonging to the class of tetracyclic triterpenes and having a structure similar to that of cholesterol (Figure 1). The human body cannot naturally produce this plant sterol; therefore, it is only available through foods and diets such as vegetable oils (rapeseed, soybean, and calabar bean oils), cereals, and other vegetables, unpasteurized milk, seeds, medicinal plants, as well as nuts and legumes which are common sources of phytosterols [5]. Stigmasterol is the main ingredient in several plant extracts, including the Chinese medicinal herb *Ophiopogon japonicas* (Maidong in Chinese) [6]. This natural agent was found to be the major ingredient isolated from the leaf extract of *Annona muricata* L. [7] and *Aegle marmelos* L. from India [8]. In addition, from the extracts of *Plectranthus scutellarioides* var. *color blaze dark star* from Indonesia, commonly called iler/miana/jawer kotok [9], we identified stem bark extracts of *Albizia gummifera* natively collected from sub-Saharan Africa and Madagascar [10], and *Combretum hypopilinum* from Nigeria [11], and extracts from *Salvadora persica* L. stems [12], as well as *Acacia nilotica* ssp. *indica* (L.) leaves from India [13] and *Neocarya macrophylla* stem bark extract from Nigeria [14]. Furthermore, stigmasterol is the main component isolated from the marine microalgae *Navicula incerta* [15], *Fritillaria roylei* root extracts (10), *Ageratum conyzoides* aerial part extracts [16], *Neocarya macrophylla* stem bark extracts [17], *Cassia sieberiana* leaf extracts [18], *Pluchea indica* root extracts from India [19], as well as from the leaf extracts of *Icacina trichantha* [20] and *Azadirachta indica* [21]. Other studies have shown high concentrations of stigmasterol in plant extracts, specifically [22] in carob (*Ceratonia siliqua* L.) seeds from Turkey and Pakistani rice (*Oryza sativa* L.) [23].

Stigmasterol is chemically named [(3S, 8S, 9S, 10R, 13R, 14S, 17R)-17-[(E, 2R, 5S)-5-ethyl-6-methylhept-3- en-2-yl]-10, 13-dimethyl-2, 3, 4, 7, 8, 9, 11, 12, 14, 15, 16, 17-dodecahydro-1Hcyclopenta[a]phenanthren-3-ol] [24]. Regarding the purification and isolation of stigmasterol from plants, several approaches have been performed as spectroscopic techniques, including NMR and MS, on the stem-bark extract of *Chrysophyllum albidum* [25]. NMR (^1^H-NMR, ^13^C-NMR, and LCMS) and mass spectroscopy (ESI–MS) were used to elucidate stigmasterol in *Solanum xanthocarpum* fruits [26].

The compound stigmasterol is employed in various chemical manufacturing processes intended to generate various semi-synthetic and synthetic components for the pharmaceutical industry [27]. Within the European Union, it is listed as a food additive under the number E499, which may be used to boost phytosterol levels in the production of foods, thereby helping to improve low-density lipoprotein cholesterol (LDL-cholesterol) levels [28]. This phytosterol is C24 alkylated cholesterol operating primarily as a common constituent of the cell membrane and playing a central role in membrane stability [13]. Increased interest is focused on essential oils, algae, and plant extracts as a source of promising bioactive compounds for drug development. There are currently around 250 plant sterols that differ in function and accessibility, but stigmasterol has become a unique compound due to its diverse pharmacological properties [29]. Indeed, this phytosterol has been widely studied for its anti-diabetic, antioxidant, anticancer, anti-inflammatory, antiviral, antiparasitic, anti-osteoarthritis, antibacterial, neuroprotective, and immunomodulatory properties.

Concerning the anti-diabetic effects of this phytosterol, previous research studies have shown that the administration of stigmasterol improves the glucose transporter type 4 (GLUT4) translocation and insulin resistance, reduces fasting glucose, and induced β-cell regeneration [30]. For its anticancer properties, recent findings on stigmasterol-rich plant extracts showed significant anticancer effects against various tumor cell lines by arresting cell cycle progression and inhibiting cell growth by regulating cell proliferation. In skin, lung, gastric, cervical, endometrial, ovarian, and breast cancers, stigmasterol acts via different mechanisms, and activities seem to be dose-dependent [20,31,32]. Furthermore, stigmasterol activated pro-apoptotic proteins, triggered PI3K/Akt, Akt/mTOR, and JAK/STAT, VEGFR-2 signaling pathways involved in numerous types of cancers and suppressed chemoresistance and enhanced inhibitory activity of some antiproliferative drugs [33,34,35,36,37,38]. Moreover, recent findings on stigmasterol-rich extract assessed antitumor cell proliferation through antiviral activities, particularly against HPV encountered in 30% of cervical cancer [7,39,40]. ADMET in silico properties revealed high permeability for human intestinal stigmasterol and the blood–brain barrier; moreover, high target protein binding stability (in vitro and in vivo) showed low toxicity of this phytosterol [38,40,41,42].

Like other phytosterols, stigmasterol also exhibits various anti-osteoarthritis properties [43]. Previous studies found that stigmasterol relieved cartilage degradation in rodent models of osteoarthritis [44]. When combined with mesenchymal stem cell secretome, stigmasterol reduces inflammation and leads to constant regeneration/repair of eroded cartilage [45,46]. This phytosterol could be considered in new therapeutic designs since it has anti-inflammatory and analgesic properties that can overcome osteoarthritis effects [47]. In fact, this bioactive sterol extracted from different natural sources plays an important anti-inflammatory and analgesic role [48]. The anti-inflammatory properties of stigmasterol were assessed in vivo, and the underlying mechanism of action was demonstrated by molecular docking analysis [49,50,51,52].

Stigmasterol-rich plant extracts exhibited effective anti-inflammatory and immunomodulatory activities in vivo [53]. It was able to ensure the decrease in the release of tumor necrosis factor-α (TNF-α), nitric oxide (NO), and pro-inflammatory cytokines, as well as the inhibition of cyclooxygenase-2 (COX-2) [54,55]. Traditional Chinese medicine values stigmasterol-containing drugs in immune inflammatory response control, such as Smilacis Glabrae Rhixoma (SGR) for the management of myasthenia gravis [56] and xiaoqinglong (XQLD) against severe coronavirus disease 2019 (COVID-19) [57].

The studies carried out have highlighted the immunomodulatory activities of stigmasterol and extracts containing this compound. Indeed, stigmasterol has been shown to up-regulate the intestinal mucosal immune response involved in inflammatory bowel disease (IBD) by activating the butyrate-PPARγ axis in colitis [53]. It also stimulated (in vivo) specific and non-specific immune responses [26,49]. Additionally, plant extracts containing stigmasterol showed potent immunomodulatory activity in vitro. It was able to reduce the release of pro-inflammatory mediators (TNF-α, NO, IL-1β, and IL-6), as well as COX-2 activity [54].

Regarding the management of neurodegenerative diseases, previous studies have reported the great interest of stigmasterol from medicinal plants. The neuroprotective activities concerned, in particular, the GABAergic mechanism and the inhibition of acetylcholinesterase mitigating acetylcholine-mediated neurotransmission alteration [58,59].

The suppression of oxidative stress provided by stigmasterol induces both antitumoral and neuroprotective activities [8,60,61]. Recently, Haque et al. [62] found that stigmasterol attenuates excitotoxicity, DNA damage, and mitochondrial dysfunction as well as decreasing ROS production. Stigmasterol enhanced the activities of antioxidant enzymes (catalase (CAT), superoxide dismutase (SOD)), and nitric oxide synthase enzymes (iNOS and nNOS), providing neuroprotective effects [63,64]. Other pathways involved in the protective effect of stigmasterol-rich extracts have been reported earlier [65,66].

Numerous works have shown the potential antimicrobial activities of stigmasterol. The latter exhibited bacteriostatic and bactericidal activities against a broad range of Gram-positive and Gram-negative bacteria [21,67,68]. Phytosterols are able to inhibit bacterial cell surface protein acts and induce bacterial membrane composition modification [69]. Previous studies, conducted on extracts containing stigmasterol, also revealed antifungal activities against specific *Candida* species [70,71] and other pathogenic fungal species [72].

Concerning the antiparasitic properties of stigmasterol, studies have been carried out to assess whether this molecule has activities against certain strains of parasites. Phytosterol treatment decreased the membrane binding of *Trypanosoma congolense* to sialic acid. However, in silico tests showed an uncompetitive inhibition of *T. congolense* sialidase [73]. In an in vitro investigation, the inhibitory effects of stigmasterol were also assessed on promastigotes and amastigotes of *Leishmania major* [74].

This review focused on a complete and up-to-date understanding of the health benefits and biological and pharmacological activities of the stigmasterol molecule, offering an extensive background to describe its mode of action (in vitro and in vivo). We are convinced that this review will provide new perspectives for further explorations of this deterrent molecule. The outcomes of this study should provide a new pharmacological approach for the use of stigmasterol as a key drug in the management of various illnesses, particularly in cancer therapy. However, toxicological studies are required to assess its safety; the analysis of such studies could also aid in the identification of many approaches for the successful development of new drugs. Moreover, a better knowledge of stigmasterol pharmacodynamics and pharmacokinetics is essential for its application in the therapeutic field and drug discovery.

## 2. Sources and Physicochemical Properties of Stigmasterol

Stigmasterol is a phytosterol found in many foods, including soybean, rapeseed, and Calabar bean oils. Furthermore, it has been isolated from some medicinal plants such as *Ficus hirta*, *Croton sublyratus*, *Eclipta prostrate*, *Eclipta alba* (L.) Hassk, *Eucalyptus globules*, *Akebia quinata*, *Desmodium styracifolium*, *Gypsophila oldhamiana*, *Parkia speciosa*, *Emilia sonchifolia*, *Aralia cordata*, and *Heracleum rapula* [75,76,77,78,79,80,81,82,83,84,85,86].

Like all phytosterols, stigmasterol presents in the form of a white powder, with a characteristic sweet odor, and is insoluble in water but soluble in alcohols. Stevens and colleagues [83] used nuclear magnetic resonance to measure the solubility limit of some biologically important sterols including stigmasterol, lanosterol, *β*-sitosterol, cholesterol, and ergosterol. The results indicate the low solubility of stigmasterol (20–25% mol) compared with other sterols studied. Knowing that stigmasterol differ from cholesterol only by their alkyl tails, the low solubility shows that differences in the structure of the tail can strongly influence the solubility of sterols.

## 3. Anticancer Properties

**Figure 2:**
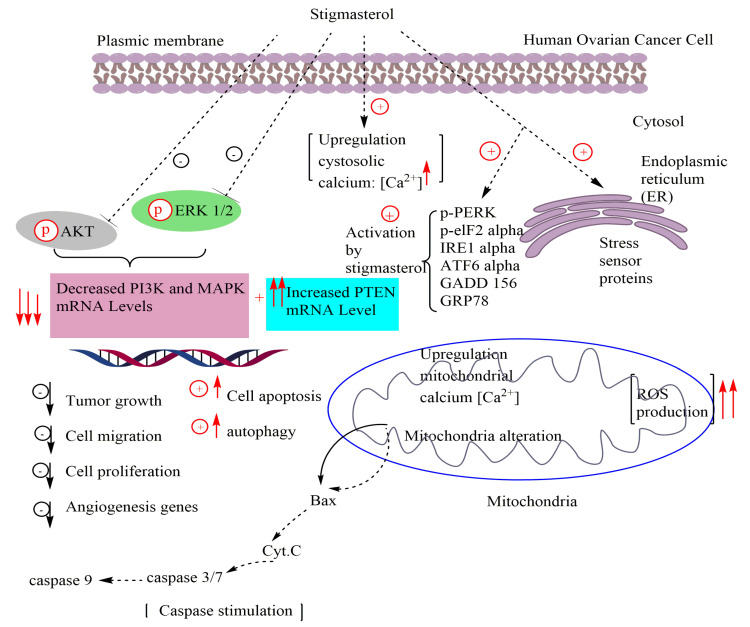
Anticancer mechanisms of stigmasterol.

Several types of investigations have revealed that stigmasterol exhibits significant anticancer properties against various tumor cell lines such as cholangiocarcinoma, hepatoma, endometrial adenocarcinoma, gallbladder carcinoma, as well as skin, gastric, cervical, breast, prostate, and ovarian cancers and can, consequently, be considered as one of the new perspectives in the pharmacological therapy of cancerous diseases (Table 1). Effectively, Bae et al. [87] evidenced that stigmasterol could be a promising molecule to prevent ovarian cancer. They showed that treatment with this phytosterol activates pro-apoptotic protein signals and stimulates the cleavage of caspase-3, caspase-9, cytochrome c, BAK, and BAX in ES2 and OV90 cells in a dose-dependent manner, resulting in the suppression of cell aggregation. In addition, stigmasterol was shown to induce cell apoptosis in ovarian cancer by leading to a significant alteration in mitochondrial function, increased calcium levels in the cytosol and mitochondria, as well as excessive ROS generation. Based on Western blot analysis, stigmasterol treatment (0, 5, 10, and 20 μg/mL) showed a significant ability to up-regulate the expression of endoplasmic reticulum–mitochondrial axis proteins and endoplasmic reticulum stress sensor proteins in ovarian cancer cells including phosphorylated eukaryotic translation-initiation factor 2α (p-eIF2α), inositol-requiring enzyme 1α (IRE1α), activating transcription factor 6α (ATF6α), phosphorylated PKR-like ER-resident kinase (p-PERK), growth arrest and DNA damage-induced-153 (GADD153), glucose-regulated protein 78 (GRP78), and phosphorylated eukaryotic translation-initiation factor 2α (p-eIF2α) stimulating cell death. Furthermore, stigmasterol arrested cell cycle progression and inhibited cell growth by regulating cell proliferation and inactivating intracellular signaling cascades, particularly the PI3K/MAPK pathway, but also decreased cell migration activity in human ovarian cancer cells [87] (Figure 2). Similarly, Li et al. [88] conducted a study using in vitro cell experiments to explore the protective effects of stigmasterol, as the main biological compound found in the Chinese herbal medicine Xiaoyao San, against ovarian cancer.

According to the molecular docking result, stigmasterol exhibits a high degree of interaction with Akt1. As evidenced by previous works, the dysregulation of PI3K/Akt signaling pathway were identified in 70% of ovarian cancers, involving PIK3CA mutations and a PTEN deletion [37]. The results of this study indicated that stigmasterol could be a potential drug of interest for the management of ovarian cancer by triggering the PI3K/Akt signaling pathway.

Based on the outcomes of wound healing assay and CCK8 assay, the action of stigmasterol could successfully suppress the growth and migration of A2780 (IC_50_ = 69.24 ± 7.31 for 24h) and SKOV3 (IC_50_ = 83.39 ± 3.75 for 24h) cells. Additionally, stigmasterol can decrease PI3K and Akt levels and increase PTEN expression in both cells studied [88].

Endometrial cancer is most commonly diagnosed in postmenopausal women and is the most frequent gynecological malignancy [33]. Nrf2 has been reported to be overexpressed in endometrial cancer tissues. In this context, Liao et al. [36] conducted a study to assess the possible chemopreventive effects of natural compounds such as stigmasterol and to elucidate its implication in inhibiting the Nrf2 signaling pathway. Based on IHC analysis, Nrf2 was identified to play a pivotal role in endometrial cancer progression and chemotherapy resistance, as Nrf2 was found to be more expressed in endometrial carcinoma tissue compared to normal endometrium and also resulted in decreased sensitivity to cisplatin. Indeed, stigmasterol increases the sensitization of endometrial cancer cells to cisplatin by suppressing Nrf2, NQO1, and HO1 expression in a dose-dependent manner. In addition, early apoptosis was found to be higher and endometrial cancer growth was inhibited when stigmasterol was combined with cisplatin. Additionally, stigmasterol reinforces the inhibitory activity of cisplatin on migration and invasion. In addition, the results showed that stigmasterol suppresses Tet1 expression and Tet1-induced hydroxymethylation, leading to suppression of Nrf2 protein expression. Therefore, the down-regulation of Nrf2 protein by stigmasterol is also mediated by the reduction in transcriptional activity [36]. These findings demonstrated that the combined treatment of stigmasterol with cisplatin could be used as a promising therapy to suppress chemoresistance in endometrial cancer via inhibition of Nrf2 pathway expression.

In order to show the anticancer properties of stigmasterol against gastric cancer, Li et al. [89] sought to investigate the effect of this compound against SNU-1 gastric cancer cells while evaluating its inhibitory power on the proliferation and colony formation of cancer cells. Undoubtedly, treatment with stigmasterol exhibited inhibitory effects on the proliferation, growth, and colony formation in SNU-1 gastric cancer cells (IC_50_ = 15 µM) as revealed by CCK-8 assay, concentration-dependent apoptotic cell death caused by mitochondrial-mediated apoptosis as observed by DAPI, and annexin V/PI staining assay confirmed by increased Bax expression and decreased Bcl-2, as evidenced by the Western blot assay. Stigmasterol has also been observed to suppress cancer cell migration and induce G_2_/M cell cycle arrest. Furthermore, this medicinal plant could suppress the expression of JAK-1, JAK-2, SATA-1, and STAT-2 and also could inhibit the phosphorylation of pJAK-1, pJAk-2, pSTAT-1, and pSTAT-2. Therefore, these findings indicated that stigmasterol targets inhibition of JAK/STAT signaling pathway, as shown by the in vitro experiment. In an effort to investigate the hypothesis that the Akt/mTOR pathway is a major molecular cascade triggered in a wide range of cancers, and its activation can lead to cancer progression [90]. In this sense, Zhao et al. [91] in their in vivo and in vitro investigations examined the anti-cancer effect of stigmasterol by inhibiting Akt/mTOR pathway in human gastric cancer cell lines SGC-7901 and MGC-803. The results indicated that stigmasterol treatment has a significant effect to inhibit the viability, cell growth, and proliferation of gastric cancer cells in a dose-dependent manner, as shown by CCK8 assay, cloning formation, and EdU incorporation assays. They also identified the underlying molecular mechanism of stigmasterol that induces autophagy and apoptosis by inhibiting the Akt/mTOR signaling pathway via down-regulated expression of both p-mTOR and p-Akt in cancer gastric cells in a concentration-dependent manner as determined by Western blot assay. Moreover, stigmasterol showed beneficial in vivo effects to inhibit tumor growth in MGC-803 and SGC-7901 cells [91]. These outcomes revealed that stigmasterol could be considered as a promising alternative for the treatment of gastric cancer.

Recently, Farshori et al. [34] assessed in vitro the anticancer potential of *Aloe perryi* petroleum ether extract (APPeE) and identified the probable mechanisms implied using human breast cancer cells (MDA-MB-231) and normal (HEK-293) cell lines. The densitometric high-performance thin-layer chromatography (HPTLC) method has provided that stigmasterol was the main biomarker isolated from *A. perryi* extract, the quantification percentage was 0.238% *w*/*w* of dried APPeE. It is evident from the findings that this extract induced cytotoxicity and apoptosis in MDA-MB-231 cells. APPeE also caused an increase in ROS production and caspase-3 and -9 protein levels. In addition, data from this investigation demonstrated overexpression of the pro-apoptotic genes Bax, p53, caspase-3 and -9, and down-regulation of the Bcl-2 gene, as determined by Western blot assay further confirm that APPeE has the potency to induce MDA-MB-231 cell death via the apoptotic pathway. Furthermore, MDA-MB-231 cells treated with increased concentrations of APPeE induced morphological alterations, as evidenced by cell morphological assay. The predominant content of stigmasterol in APPeE could provide it with promising anti-cancer benefits in the future to treat breast cancer [34].

Furthermore, Omran et al. [38] performed a study to evaluate the possible anti-tumor effect of a combination of stigmasterol and sorafenib on angiogenesis, proliferation, and apoptosis signaling implicated in promoting breast cancer progression using the MCF-7 and MDA-MB 231 cell lines. A combination of sorafenib and stigmasterol down-regulated the proliferative marker Ki-67. Significant anti-angiogenic activity was also observed with both compounds independently. Levels of p-ERK and its downstream transcription factor, NF-κB, were both decreased. Both drugs also caused a reduction in the expression of the angiogenic mediator, VEGF-A, and its receptor, VEGFR-2. In addition, both stigmasterol and sorafenib promoted caspase-3 activity and down-regulated levels of the anti-apoptotic protein Bcl-2. The cytotoxicity of stigmasterol was low. Hence, the association of stigmasterol and sorafenib could prove beneficial in the treatment of breast cancer [38].

Using molecular docking simulation and an in silico absorption, distribution, metabolism, excretion, and toxicity (ADMET) approach, Spriha et al. [42] in their recent investigation aimed to identify some selected agents isolated from the multipotent plant *Bergenia ciliate* able to inhibit molecular targets involved in breast cancer, including PR (progesterone receptor), ER-α (estrogen receptor-α), EGFR (epidermal growth factor receptor), and HER2 (human epidermal growth factor receptor 2). Among the fifteen components analyzed, the results obtained from the molecular docking showed that stigmasterol has stronger binding affinities −9.4, −9.4, −10, and −8.8 kcal/mol towards ER-α, PR, HER2, and EGFR, respectively. Additionally, stigmasterol extracted from *Bergenia ciliata* showed hydrophobic interaction with amino acid residues of ER-α (Leu346, Ala350, Leu354, Trp383, Leu384, Leu387, Leu525, Leu536, and Leu539), PR residues (Leu715A, Leu718A, Phe778A, Trp755A, Met759A, Phe794A, Leu797A, Met801A, Ile896A, and Cys891A), HER2 residues (Leu726, Val734, Ala751, Lys753, Leu800, Cys805, Leu852 and Phe1004), and EGFR residues (Leu718, Val726, Ala743, Lys745, Leu792, Met793, Cys797, and Leu844). In addition, in silico ADMET properties revealed that stigmasterol was identified as a substrate for P-glycoprotein and CYP3A4. It demonstrated high permeability for human intestinal absorption and Caco-2 cells, as well as high blood–brain barrier permeability, and showed no carcinogenicity [42]. Based on these data, stigmasterol showed significant properties to be an excellent compound in the search for new multi-targeted drugs to prevent breast cancer. On the other hand, using human hepatocarcinoma (HepG2) cells, stigmasterol extracted from the marine microalgae *Navicula incerta* revealed potent anti-proliferative activity and toxicity against these cells in a dose-dependent manner. This compound has been shown to arrest the cell cycle and markedly stimulate the caspase cascade through the intrinsic apoptotic pathway, thus leading to cell death. Indeed, the expression of Bax, p53, and caspase-8, 9 are up-regulated, but XIAP genes and Bcl-2 are down-regulated in a dose-dependent manner, as determined by Western blot assay. Furthermore, after 24 h of treatment at different concentrations (5, 10, and 20 μM), stigmasterol was found to induce morphological transformations, increase apoptotic cell numbers and lead to DNA damage in HepG2 cells, as demonstrated by annexin V staining and cell cycle analysis Hoechst staining. These data suggest that stigmasterol could be a potential candidate to protect against liver cancer [32].

Concerning skin cancer, 99% pure stigmasterol isolated from *Azadirachta indica* was tested to assess its possible chemopreventive potential against skin carcinogenesis induced by 7,12-dimethylbenz[*a*]anthracene (DMBA) and croton oil in mice. Ali et al. [20] revealed from their results that oral administration of stigmasterol (200 and 400 mg/kg three times/week for 16 weeks) markedly reduced tumor size and the cumulative number of papillomas, and increased the average latency period (13.10 ± 0.76). In order to examine oxidative stress and biochemical enzymes in treated mice, they found that stigmasterol decreases the levels of serum enzymes, including alanine aminotransferase (ALAT), aspartate aminotransferase (ASAT), alkaline phosphatase (ALP), and bilirubin. Additionally, stigmasterol was able to increase the activity of glutathione (GSH), SOD and CAT, as well as suppress elevated levels of lipid peroxide and DNA damage. Furthermore, in stigmasterol-treated animals, histological examination showed evidence of tumor, acanthosis, and hyperkeratosis, but to a lesser degree than in control animals. These findings showed that stigmasterol exerts antigenotoxic potential due to its potency against oxidative stress and could be used as a bioactive compound to prevent skin cancer [20].

Concerning the exploration of the anti-tumor effect of stigmasterol against Jab1 in gallbladder cancer cells, Pandey et al. [92] in their investigation clearly showed that this compound induces gene modulations via the down-regulation of the Jab1 gene and the up-regulation of p27 expression that could be produced by the mitochondrial apoptosis signaling pathway. As evidenced by Hoechst staining, annexin V staining, and cell cycle analysis, stigmasterol was able to induce cell apoptosis and DNA damage, as well as activate caspase-3. Therefore, these outcomes provide compelling evidence suggesting that stigmasterol has the capability to be considered as an anti-cancer drug against Jab1 in gallbladder cancer [92].

On the other hand, the aim of the study performed by Ghosh et al. [93] was to investigate the anticancer effect of stigmasterol isolated from the aerial parts of *Bacopa monnieri* L. against Ehrlich ascites carcinoma (EAC) in Swiss albino mice. Stigmasterol showed chemopreventive effects by decreasing tumor volume, packed cell volume, and viable cell number, as well as increasing mean survival time, thereby improving the lifespan of mice with EAC tumors. Regarding oxidative stress, stigmasterol treatment at doses of 5 and 10 mg/kg to mice with EAC decreased lipid peroxidation levels by 49.0% and 56.1%, respectively, compared with the EAC control group (*p* < 0.01). Furthermore, this phytosterol caused an increase in GSH levels and SOD and CAT activities, demonstrating its ability to suppress carcinoma-mediated intracellular oxidative stress in mice. Hematological analysis indicated that stigmasterol exhibits protective activity on the hematopoietic system; it increased the levels of hemoglobin, platelets, and red blood cell count, but reduced the number of white blood cells with an increase in monocytes and lymphocytes and a decrease in neutrophils in treated mice compared with normal animals in a dose-dependent manner. Moreover, stigmasterol also increased LDH activity in ascitic fluid and caused a decrease in membrane microviscosity. These findings showed that stigmasterol could be a promising drug for Ehrlich’s ascites carcinoma [93].

Kangsamaksin et al. [41] have investigated (in vitro) the impacts of stigmasterol on human umbilical vein endothelial cells (HUVECs) and its anti-tumor properties (in vivo) against human cholangiocarcinoma (CCA) cells. The results demonstrated that stigmasterol exhibits potent toxic activity on HUVECs (IC_50_ = 21.1 ± 2.1 μM) by significantly inhibiting cell proliferation, migration, viability, morphogenesis, and capillary network formation. As shown by expression analyses, stigmasterol markedly disrupted (in vitro) angiogenesis by down-regulating TNF-α and inhibiting VEGFR-2 signaling levels, including phosphorylated forms of Akt, Src, PCL, Akt, and FAK in vitro, but showed no effect on IL-6 and CXCL-8. In the in vivo experiment, stigmasterol did not show signs of liver toxicity and showed no significant effect on levels of systemic and liver injury markers. However, stigmasterol significantly suppressed tumor growth, tumor angiogenesis, and macrophage recruitment in CCA xenograft models by inhibiting the production of inflammatory cytokines. These data proved the important role of stigmasterol in suppressing tumor growth and endothelial morphogenesis through the TNF-α-VEGFR-2 axis [41].

Moreover, the stigmasterol compound isolated from the stem bark of *Aglaia simplicifolia* was found to exert cytotoxic effects against HeLa cervical cancer cells with an IC_50_ of 26.42 µM. Nevertheless, ergosterol peroxide agents showed stronger activity in reducing the viability of Hela cells. The IC_50_ value was 0.80 µM, which may be due to its ability to induce mitochondrial alteration, apoptosis, and ROS production [35].

Intending to examine the chemopreventive effect of stigmasterol against lung cancer, Dong et al. [31] showed that stigmasterol exhibits an anticancer effect against lung cancer by promoting apoptosis and inhibiting proliferation by modulating retinoic acid-related orphan receptor C (RORC, a transcription factor that binds to DNA, is a family of receptors for nuclear orphans. It has aroused a lot of interest since it has an essential regulatory effect on cell proliferation, chemoresistance, and metastasis of different types of malignancies, identified as a potential target of stigmasterol on lung cancer. It was found that overexpression of RORC induces, to some extent, a reversal of the inhibitory activity of stigmasterol on lung cancer cells. Additionally, stigmasterol treatment (5, 10, and 20 µg/mL) resulted in a significant increase in caspase-3 and caspase-9 transcriptional levels compared to the control group [31].

In contrast, stigmasterol as a major component of Saw Palmetto berry extract (SPBE) was tested (in vitro) to show its antitumor activity against human prostate cancer cells DU-145. The results revealed that stigmasterol inhibits carcinoma development by decreasing p27 and p21 protein expression and also suppresses prostate cancer growth by increasing p53 protein expression as evidenced by Western blot analysis. This phytosterol may have positive effects on prostate cancer [94].

In order to find an alternative therapy for the management of cervical cancer, most often induced by human papillomavirus (HPV: representing approximately 30% of all cancers associated with an infectious microorganism) [39], Salaria et al. [40] examined the ability of natural bioactive compounds isolated from Himalayan herbs, including stigmasterol, to inhibit HPV-18 E1 protein (1R9W). As demonstrated by molecular docking, stigmasterol extracted from *Berberis aristata* showed high binding affinity with a docking score of -8.7 kJ/mol. Using ADMET screening, this natural drug was found to be one of the best phytoconstituents able to inhibit HPV-18 (1R9W protein). Additionally, stigmasterol showed high stability inside the binding pocket of the target protein 1R9W, as revealed by MD simulations data. Therefore, it can be used as a potential antiviral agent to prevent HPV [40].

From another study, stigmasterol isolated from *Annona muricata* leaves was found to exert significant cytotoxic activity on Vero and Hela cancer cells, showing a high selective index at different concentrations, IC_50_ values were 173.8 and 11.58 µg/mL, respectively. As revealed by molecular docking analysis, this substance also displayed substantial interaction energy values against the target protein, vaccinia virus H1 (VHRV)-related phosphatase, decreasing Hela cancer cell proliferation (RCSB PDB ID: 3F81) with a binding energy value of −6.6 (kcal/mol) and an inhibitory constant (Ki) of 14.34816 M. Accordingly, this isolated agent has the potential to be used to produce medicine to cure cervical cancer [7].

**Table 1:** Anti-cancer effects of stigmasterol.

Cell Lines	Key Results	Ref
A2780 human ovarian cancer cell line SKOV3 human ovarian cancer cell line	A2780 cells (IC_50_ = 69.24 ± 7.31 for 24 h) SKOV3 cells (IC_50_ = 83.39 ± 3.75 for 24 h) Inhibited cell migration in A2780/SKOV3 cells Inhibited cell proliferation in A2780/SKOV3 cells Down-regulated the expression of p-PI3K/p-Akt protein levels Up-regulated the expression of PI3K/Akt/PTEN protein levels in SKOV3 cells Reduced the levels of PI3K and Akt in A2780/SKOV3cells Increased the expression of PTEN in A2780/SKOV3cells	[88]
Human gastric cancer cell line SGC-7901, MGC-803, and normal GES-1 cell line	Inhibited cell proliferation in SGC-7901/MGC-803 cells Induced apoptosis and autophagy in vitro Blocked Akt/mTOR signaling pathway Suppressed tumor growth of gastric cancer in vivo	[95]
Human ovarian cancer cells	Inhibited development of ES2 and OV90 cells Induced cell apoptosis Suppressed cell migration Inhibited angiogenesis genes Increased ROS production Increased calcium levels in the cytosol and mitochondria Increased mitochondrial depolarization Stimulated cell death Activated the ER-mitochondrial axis Inactivated PI3K and MAPK signal cascades	[87]
Human endometrial cancer cell lines, Ishikawa and SPEC2	Inhibited Nrf2/NQO1/HO1 expression in endometrial cancer cells Suppressed Nrf2-ARE activity when combined with cisplatin Enhanced early apoptosis when combined with cisplatin Inhibited Tet1 expression/Tet1-induced hydroxymethylation Enhanced the inhibitory effect of cisplatin on migration and invasion	[36]
Human breast cancer cells (MDA-MB-231) and normal (HEK-293) cell lines	IC_50_ = 24.5 μg/mL Induced strong cytotoxic effects on MDA-MB-231 cells Increased ROS production Activated caspase-3 and -9 Induced apoptosis in MDA-MB-231 cells Increased levels of p53, Bax, caspase-3 and -9 Declined Bcl-2 gene expression Induced MDA-MB-231 cell death via apoptotic pathway	[34]
Molecular docking simulation In silico ADMET approach Molecular targets of breast cancer: ER-α, PR, HER2, and EGFR	Inhibited molecular targets of breast cancer: PR/ER-α/EGFR/HER2 Exhibited stronger binding affinities towards PR/ER-α/EGFR/HER2 Showed hydrophobic interaction with amino acid residue of PR/ER-α/EGFR/HER2 Identified as a substrate for P-glycoprotein and CYP3A4 Demonstrated high permeability for human intestinal absorption and Caco-2 cells Showed high blood–brain barrier permeability Showed no carcinogenicity	[42]
Human breast cancer cell lines MCF7 and MDA-MB-231	Showed low cytotoxicity Significantly reduced levels of p-ERK/NF-Kb/VEGF/VEGFR-2 (*p* ≤ 0.05) Reduced Ki67 levels when combined with sorafenib (*p* ≤ 0.05) Markedly decreased VEGFR-2 mRNA gene expression Increased caspase-3 activity Decreased Bcl2 levels	[38]
Human gastric cancer cell line SNU-1, GES-1 normal cell line	IC_50_ = 15 µM Inhibited gastric cancer growth Inducted mitochondrial-mediated apoptosis Inhibited cancer cell migration Induced cell apoptosis Triggered G_2_/M cell cycle arrest in a dose-dependent manner Inhibited the JAK/STAT signalling pathway Exhibited minimal anticancer effects on the normal GES-1 cells Suppressed the phosphorylation of pSTAT1/pSTAT 2/pJAK1/pJAk2	[89]
Lung cancer cell lines	Inhibited the proliferation Promoted the apoptosis Modulated retinoic acid-related orphan receptor C (RORC) Increased levels of caspase-3 and caspase-9	[31]
Human hepatoma HepG2 cells	Showed potent cytotoxicity against HepG2 cells in a dose-dependent manner Up-regulated the levels of Bax, p53 Down-regulated the levels of Bcl-2 Induced apoptosis: caspase-8, 9 Increased apoptotic cell numbers Induced morphological changes Induced DNA damage	[32]
DMBA croton oil induced skin carcinoma	Reduced tumor size and cumulative number of papillomas Significantly increased the average latency period Decreased the levels of ASAT/ALAT/ALP Significantly increased glutathione (GSH)/superoxide dismutase (SOD)/catalase (CAT) Significantly inhibited high levels of lipid peroxide and DNA damage	[20]
Human gall bladder cancer cells	Up-regulated p27 expression gene Down-regulated Jab1 expression gene Activated caspase-3 Induced apoptosis Increased apoptotic cells and DNA	[92]
Ehrlich Ascites Carcinoma mice	Decreased tumor volume Decreased packed cell volume and viable cell count Increased mean survival time Increased life span of EAC tumor bearing mice Decreased the levels of lipid peroxidation Increased the levels of GSH/SOD/CAT Increased LDH activity in ascitic fluid Decreased membrane microviscosity Activated protein phosphatase 2A by ceramide Protected the heamoto-poietic system Increased the hemoglobin content/RBC count Reduced WBC count Increased platelet count	[93]
Human umbilical vein endothelial cells (HUVECs) Human cholangiocarcinoma (CCA) cells	IC_50_ = 21.1 ± 2.1 μM Suppressed cell viability, migration, and morphogenesis on HUVECs, but not CCA cells Significantly reduced the transcript level of TNF-α Decreased levels of VEGFR-2/Src/Akt/PCL/FAK Disrupted tumor angiogenesis (in vivo) Reduced the growth of CCA cells (in vivo) Decreased CD31-positive vessel content Decreased macrophage recruitment	[41]
HeLa cervical cancer cells	IC_50_ = 26.42 µM Exhibited cytotoxic effects Reduced cell viability	[35]
Human prostate cancer cells DU-145	Inhibited prostate cancer growth Increased p53 protein expression Inhibited carcinoma development Decreased p21 and p27 protein expression	[94]
Molecular docking In silico Approach (ADMET screening) Human papillomavirus type (HPV-18 E1) protein (PDB ID: 1R9W) MD simulations	Showed high binding affinity: docking score of −8.7 kJ/mol Inhibited HPV-18 (1R9W protein) Showed high stability inside the binding pocket of 1R9W	[40]
Cervical cancer cells (HeLa) and Vero cells (L-929) In silico molecular docking	HeLa cells: IC_50_ = 11.58 µg/mL Vero cells: IC_50_ = 173.8 µg/mL Showed great in silico molecular docking activity Exhibited in vitro anticancer activity Exhibited potent cytotoxic activity at a concentration of 100 μg/mL Strongly occupied the active location of 3F81 Exhibited substantial interaction energy values against the protein target vaccinia H1-related (VHR) phosphatise Reduced the proliferation of cervical cancer cells (RCSB PDB ID: 3F81)	[7]

## 4. Anti-Osteoarthritis Effects

Osteoarthritis (OA) is nowadays one of the most widespread chronic joint diseases in the world; its incidence and prevalence increase with age, and it affects the majority of people over 65 years of age. It is one of the main musculoskeletal causes of reduced mobility in the elderly [43].

Previous research has shown that phytosterols exert a variety of biological actions, including anti-osteoarthritis properties (Table 2). In this sense, Chen et al. [44] assessed the in vivo activities of stigmasterol on cartilage degradation in a rabbit model of OA. The result showed that this compound was able to significantly inhibit cartilage degradation and the progression of cartilage alteration in a rabbit anterior cruciate ligament transection (ACLT) model, as evidenced by histological and morphological examination. In addition, stigmasterol treatment resulted in markedly decreased expression of matrix metalloproteinases (MMP)-1, MMP-3, MMP-13, and increased tissue inhibitors of metalloproteinase (TIMP-1). Indeed, it has been noticed that the expression of TIMP-1 decreases with cartilage degradation. Therefore, the equilibrium between MMPs and TIMPs was altered. Because the imbalance between MMPs and TIMPs was enhanced with progressive cartilage degradation, which reinforced the prominent role of TIMPs/MMPs in the OA process [44].

In order to develop a novel therapeutic approach to alleviate OA, Sampath et al. [45] conducted an in vivo study to examine the effect of the combination of mesenchymal stem cells (MSCs) and stigmasterol in a monosodium-iodoacetate (MIA)-induced experimental OA in rats. The results demonstrated that the combination treatment led to consistent regeneration/repair of eroded cartilage in the femoral condyles and also in the trochlear groove, highlighting the importance of combined treatment over individual therapies in rats with OA. Furthermore, the findings indicated that the combined intake of placental-derived MSCs (hPMSCs) and stigmasterol was the most potent in remedying OA damage, with simultaneous regeneration and repair, thus yielding precious insights into the in situ use of a combination of hPMSCs and stigmasterol to improve the repair and regeneration of cartilage in OA. Data from this study suggest that combination therapy will have a synergistic benefit on generalized cartilage damage in OA and lead to better disease management, but the mechanisms implicated remain unclear [45].

Similarly, Sampath et al. [46] attempted in their investigation to test in vitro the possible beneficial effects of stigmasterol with the MSC-condition medium (MSC-CM). The results showed a significant decrease in MMP-3, MMP-13, and ADAMTS-5, as well as a significant increase in the expression of collagen type II, alpha 1 (COL2A1) was found to mimic OA in vitro in primary rat articular chondrocytes, as evidenced by immunofluorescence and qRT-PCR. In fact, the combined therapy (MSC cm and stigmasterol) elicited a greater anticatabolic effect by suppressing IL-1β-induced NF-κB activation, as indicated by insignificant phosphorylation of p65 and IκBα subunits [46].

In order to study the impact of stigmasterol on metalloproteinases and inflammatory mediators produced by chondrocytes, Gabay et al. [96] used mouse and human chondrocytes incubated for 18 h with or without IL-1β. Therefore, they proved that stigmasterol effectively suppresses key pro-inflammatory (IL-6) and matrix degradation mediators including MMPs-3, MMPs-13, A Disintegrin and Metalloproteinase with thrombospondin motifs (ADAMTS)-4, and prostaglandin E_2_ (PGE_2_) involved in OA-induced cartilage degradation, mainly by suppressing the IL-1β-induced NF-κB pathway. Aggrecan and type II collagen mRNA levels were markedly diminished [96].

In the same context, Mo et al. [47] and colleagues sought to investigate the impact and mechanism of sterol regulatory element-related transcription factor 2 (SREBF2) on IL-1β-induced chondrocytes in the presence of stigmasterol. The results indicated that stigmasterol showed no significant activity on the viability of ATDC5 cells. It has the ability to attenuate IL-1β- and ferroptosis-induced ATDC5 cell injury mediated by SREBF2, and strengthened the inhibitory effect of ferroptosis inhibitors on IL-1β-induced ATDC5 cell injury. These findings suggested that stigmasterol alleviated IL-1β-induced chondrocyte injury through regulation of ferroptosis via SREBF2 down-regulation. This phytosterol could be promising as a new approach in the treatment of OA [47].

On the other hand, Zhang et al. [97] proved that stigmasterol was identified as a potential target in *Radix Achyranthis Bidentatae* (RAB) as evidenced by the network pharmacological approach and maybe the most important component in the treatment of RAB-mediated OA. RAS has been shown to delay the development of OA and minimize associated pathological damage. Moreover, the findings of the enrichment assay showed that the major pathways implicated in the therapeutic process are IL-17 signaling pathway, PI3K/AKT pathway, viral infection, and cell apoptosis. Moreover, active compounds in RAB, including stigmasterol, may have the potential to minimize chondrocyte apoptosis and also protect the synovial lining and joint cartilage to control OA progression.

It is essential to highlight that there are 63 target genes related to the treatment of OA by RAB, of which IL-6, EGFR, MAPK8, CCND1, CASP3, ESR1, VEGFA, and MYC represent the pivotal target genes [97].

Additionally, anti-arthritic activity and toxicity investigations in vitro and ex vivo by the chick chorioallantoic membrane (CAM) assay have proven that stigmasterol exhibits a stronger response of anti-inflammatory dysfunction correlated to the arthritis disease activity. Furthermore, the CAM assay displays anti-arthritic activity via the mTORC1 signaling pathway and deactivates certain inflammatory mediators, which improves cellular healing and joint health. The LC_50_ toxicity value was 87.7 µg/mL. Moreover, CAM was found to be effective in decreasing the inflammation induced in synovial joint cartilages at an increased dose of 20 µg/mL. These data suggest that stigmasterol may be considered a potential beneficial agent in the management of OA [98]. In the same context, stigmasterol existing among various active components of Shenjin Huoxue Mixture (SHM) exhibited potential pharmacological effect against OA through the molecular mechanisms involving the Toll-like receptor (TLR) cascade pathway and an IL-6 signaling pathway [99].

Furthermore, stigmasterol was one of the main ingredients found in Xiao Huoluo Pills (XHLP), it has been revealed to exert beneficial effects on cartilage degeneration of knee OA, as demonstrated by molecular docking and bioinformatics analysis. This active biomolecule could be related to six targets, namely, NCOA2, PGR, PTGS1, PTGS2, RXRA, and NR3C2. These primary target genes are linked to signaling pathways involved in cartilage degeneration in knee OA, including the PI3K-Akt signaling pathway and the TNF-α signaling pathway [100].

**Table 2:** Anti-osteoarthritis effects of stigmasterol.

Experimental Approaches	Key Results	References
Experimental rabbit osteoarthritis (OA) model induced by anterior cruciate ligament transection (ACLT)	Reduced cartilage damage progression Inhibited cartilage degradation Down-regulated MMP-1/MMP-3/MMP-13 expression Up-regulated TIMP-1 Regulated the balance between MMPs and TIMPs	[44]
Monosodium-iodoacetate (MIA)-induced rat model of OA	Significantly corrected the OA lesions Enhanced cartilage repair and regeneration Provided a suitable milieu in situ Aided in cartilage regeneration Activated the resident progenitors Enhanced tissue repair and healing	[45]
Interleukin-1 beta (IL-1β)-induced inflammation in rat chondrocytes	Reduced iNOS/IL-6/MMP-3/MMP-13/ADAMTS-5 expression Increased COL2A1 expression Inhibited IL-1β-induced NF-κB activation	[46]
A model of newborn mouse chondrocytes and human OA	Inhibited pro-inflammatory/matrix degradation mediators Decreased MMP-3/MMP-13/ADAMTS-4/PGE_2_ Reduced Type II collagen and aggrecan mRNA levels Counteracted the IL-1β-induced NF-κB pathway	[96]
Interleukin (IL)-1β-induced chondrocytes	Revealed no significant effect on the viability of ATDC5 cells Reduced IL-1β-induced ATDC5 cell damage and ferroptosis Enhanced the inhibitory effect of ferroptosis inhibitors Down-regulated SREBF2	[47]
Molecular docking analysis Network pharmacological approach and methods	Retarded the development of OA Minimized the associated pathological damage Involved in cell apoptosis/PI3K/AKT/IL-17 signaling pathways/viral infection Reduced chondrocyte apoptosis Protected the synovial lining and cartilage Targeted IL-6/EGFR/MAPK8/CCND1/CASP3/ESR1/VEGFA/MYC	[97]
Chick chorioallantoic membrane (CAM) assay	LC_50_ = 87.7 µg/mL Showed anti-arthritic activity by mTORC1 signalling pathway Deactivated certain inflammatory mediators Increased cell recovery and joint health Reduced the inflammation caused in cartilages of synovial joint Inhibited the formation of the IL and TNF-α pathways	[98]
Network Pharmacology Approach Thin-Layer Chromatography Analysis Enrichment analysis	Exhibited potential pharmacological effect against OA Involved Toll-like receptor (TLR)/IL-6 signaling pathways	[99]
Molecular docking analysis Bioinformatics analysis	Exerted beneficial effects on cartilage degeneration Targeted NCOA2/PGR/PTGS1/PTGS2/RXRA/NR3C2 Involved PI3K-Akt/TNF signaling pathways	[100]

## 5. Anti-Inflammatory Effects

Stigmasterol appears to play an important role in diminishing inflammation, which may be due to the presence of chemical compound precursors that can limit inflammatory processes. Table 3 summarizes investigations that have demonstrated the anti-inflammatory role of stigmasterol.

In an effort to identify the molecular mechanisms underlying the anti-inflammatory activity of stigmasterol in the management of rheumatoid arthritis (RA) caused by increased generation of pro-inflammatory cytokines, Khan et al. [101] conducted a study using collagen-induced arthritis (CIA)-induced inflammation in rats. They have indicated that stigmasterol increases the development of severe clinical symptoms in CIA rats compared to the control group. The beneficial changes were related to a lowered level of joint destruction and enhanced histological damage. Additionally, stigmasterol therapy markedly inhibited the expression of pro-inflammatory mediators, including iNOS, IL-6, IL-1β, COX-2, and TNF-α, and up-regulated the expression of anti-inflammatory cytokines such as IL-10 via negative regulation of p38MAPK expression and NF-kBp65 (suppression of p-IKB-α activation) in the joints.

In their investigation, Sampath et al. [46] tested in vitro the possible anti-inflammatory effects of stigmasterol with MSC-condition medium (MSC-CM) to alleviate IL-1β-induced inflammation in rat chondrocytes. As evidenced by immunofluorescence analysis, a considerable decrease in iNOS and IL-6 expression was observed in rat chondrocytes from the treated group. Moreover, the combined therapy (MSC cm and stigmasterol) generated a greater anti-inflammatory activity by blocking IL-1β-induced NF-κB activation, as reflected by the insignificant phosphorylation of p65 and IκBα subunits [46].

The dimethylbenzene-induced ear edema test was used to assess the anti-inflammatory potency of stigmasterol. Stigmasterol was found to inhibit ear edema in mice in a dose-dependent manner, with a percentage inhibition of 50.34% at 30 mg/kg. This bioactive compound exhibited significant anti-inflammatory action, which was consistent with the evidence presented in the second (inflammatory) phase (*p* < 0.05) of the formalin test, suggesting that the analgesic action of stigmasterol might be in part induced by its anti-inflammatory effect [48].

Recently, Morgan et al. [51] aimed to investigate the anti-inflammatory properties of stigmasterol in mice and its underlying mechanism of action using carrageenan-induced peritonitis and arachidonic acid-induced paw edema. Therefore, stigmasterol exhibited potent anti-inflammatory action, by decreasing arachidonic acid-induced paw edema and the leukocyte infiltration in the peritonitis test. As demonstrated by molecular docking analysis, this phytosterol could interact with glucocorticoid receptors and also RU-486 inhibited the effect of stigmasterol in the acetic acid abdominal torsion test, suggesting that the mechanism of action of stigmasterol is probably the involvement of glucocorticoid receptors [51].

From another investigation, the systematic administration of stigmasterol in rodents resulted in the suppression of the major local and systemic features of TPA-induced contact dermatitis by inhibiting the expression of pro-inflammatory cytokines. Additionally, this natural sterol was able to lower the weight of the ears, whereas a measurement of the edema revealed a considerable suppression of the cutaneous inflammatory reactions. A notable outcome in this study was the down-regulation of serum TNF-α levels by stigmasterol [49].

On the other hand, in order to examine the possible anti-inflammatory properties of chemical compounds isolated from *Petiveria alliacea*, Olajubutu et al. [52] in their investigation indicated that stigmasterol was the main agent among other identified chemical compounds and reported that stigmasterol from the *n*-hexane fraction and ethanolic extract of *P. alliacea* inhibits the production of pro-inflammatory proteins. Interestingly, it exhibited the best binding energy (6.5 kcal/mol) against TNF-α. It has also proven to be an effective molecule against the COX-2 enzyme. The maximal percentage of inhibition of rat paw edema (79.86 ± 0.16%) was achieved at 5% within 4 h of treatment. Additionally, based on quantum chemical calculations, stigmasterol has been shown to be potentially beneficial for use as a topical anti-inflammatory drug, also possessing greater potential due to its enhanced electrophilic index.

Similarly, intraperitoneal administration of hexane (100 and 300 mg/kg) and hydroethanolic (100 mg/kg) fractions of ripe fruits of *Solanum lycocarpum* exerted strong anti-inflammatory activity and led to a significant suppression of carrageenan-induced paw edema in 4 and 6 h after induction of inflammatory signal. Stigmasterol was the main constituent detected from the fractions obtained and could be related to these activities [50].

Stigmasterol, as the main component of *Critonia aromatisans* hexanic extract, exhibited effective anti-inflammatory activity (in vivo). It was able to decrease macrophage release of TNF-α, NO, COX-2, and pro-inflammatory cytokines such as IL-1β and IL-6, without any alteration in macrophage viability [54].

Based on the results of the study conducted by Sharif et al. [55], stigmasterol, which might be one of the main chemical compounds of *Aerva lanata* ethyl acetate extract, improved remarkably (*p* < 0.001) the number of inflammatory cells in the blood and bronchoalveolar lavage fluid. It has been reported that ethyl acetate extract of *A. lanata* decreases (*p* < 0.001) the level of inflammatory modulator TNF-α and IgE antibodies. *A. lanata* reduced (*p* < 0.001) IL- 4, IL-5, IL-13, and increased (*p* < 0.001) the expression levels of AQP1 and AQP5 in allergic asthmatic mice [55].

In a recent study, He et al. [102] investigated the anti-inflammatory properties of phytosterols using LPS-stimulated RAW264.7 cells and LPS-induced acute lung injury in C57BL/6J mice. The results strongly indicated that phytosterols provide anti-inflammatory benefits by activating the LXRs/ABCA1 signaling pathway and altering the activation of the TLR4/NF-κB pathway [102]. Additionally, stigmasterol down-regulated the expression of inflammatory factors, including IL-6, IL-1β, and TNF-α, in dextran sodium sulfate (DSS)-treated mice [53].

## 6. Immunomodulatory Effects

According to the studies conducted, stigmasterol could be a pivotal agent due to its immunomodulatory effect (Table 4). In an effort to demonstrate the therapeutic potential of stigmasterol as an immunomodulatory agent in IBD, Wen et al. [53] have conducted a study using a DSS-induced colitis model to explore the possible role of stigmasterol against IBD and provide more detailed information on its mechanisms of action. It is essential to underline that the immune response of the intestinal mucosa has been reported to be associated with IBD, and that an imbalance between T helper 17 cells (Th17) and regulatory T cells (Treg) may be involved in the pathogenesis and progression of this intestinal disorder. In vivo experiments indicated that stigmasterol appreciably reduces DSS-induced inflammatory responses by regulating the Treg/Th17 balance. Indeed, stigmasterol significantly up-regulated the expression of IL-10 and TGF-β, but down-regulated the expression of IL-17A, as revealed by flow cytometry analysis. These results strongly suggest that stigmasterol both suppresses the Th17 cell response and promotes Treg-cell development in mice with DSS-induced colitis. Additionally, stigmasterol administration resulted in a higher generation of gut microbiota-derived short-chain fatty acids (SCFAs), specifically butyrate in the faeces of DSS-induced colitis mice. From a mechanistic standpoint, butyrate activated peroxisome proliferator-activated receptor gamma (PPARγ) and reprogrammed energy metabolism, thus promoting Treg differentiation and inhibiting Th17 differentiation, as evidenced by molecular docking. These findings indicate that PPARγ activation by butyrate restores Treg/Th17 cell balance, which could be a potential strategy by which stigmasterol alleviates IBD [53].

Khanam et al. [26] performed an in vivo study examining the possible immunomodulatory effects of stigmasterol from *Solanum xanthocarpum* fruit aqueous extract on cyclophosphamide-induced immunosuppression and the result revealed better protection by recording improvement in various hematological parameters and a neutrophil adhesion assay that stimulated the non-specific immune response [26]. IgE-mediated allergic reaction is described as a form of immunological process. Stigmasterol could exert an immunomodulatory effect on ovalbumin-induced airways. In fact, at 10–100 mg/kg, it caused a decrease in the proliferation of lymphocytes, eosinophils, and monocytes, as well as a decrease in the perivascular, peribronchiolar, and alveolar infiltration of inflammatory cells. In addition, stigmasterol exhibited inhibitory effects by significantly reducing VCAM-1 expression at 50 and 100 mg/kg as well as a significant decrease in serum levels of OVA-specific immunoglobulin E (OVA sIgE) [103].

In contrast, stigmasterol, as the main component of *Critonia aromatisans* leaf extracts, showed potent immunomodulatory activity in vitro. It was able to reduce the macrophage release of TNF-α, NO, COX-2, and the cytokines IL-1β and IL-6, without any alteration in macrophage viability [54].

Based on Chinese Medicine Network Pharmacology, *Smilacis Glabrae Rhixoma* (SGR) was used to explore its potential target and signaling pathway for the management of myasthenia gravis. Stigmasterol was found to be one of the main active components of SGR. As evidenced by Kyoto Encyclopedia of Genes and Genomes (KEGG) pathway assay, SGR could play a pivotal role in the treatment of myasthenia gravis and has immunomodulatory properties. The associated targets of SGR in the treatment of myasthenia gravis have been shown to focus primarily on the PI3K-Akt signaling pathway, the TNF-α and IL-17 signaling pathways, and Th17 cell differentiation, which are conventional pathways for the pathogenesis and development of myasthenia gravis. These data reflect the multi-target and immune response of SGR in treating this disease and could provide further pharmacological activities [56].

Furthermore, phytosterols, including stigmasterol from xiaoqinglong decoction (XQLD) extract, have demonstrated immunomodulatory properties against COVID-19. As demonstrated by biological network analysis, stigmasterol might be a key ingredient of XQLD, whereas STAT3, CCL2, MAPK1, FOS, VEGFA, CASP3, IL-6, MAPK3, MAPK8, and CASP8 may represent potential drug targets. Molecular docking analysis results have revealed high affinity of the target proteins of the host cells with critical ingredients, including stigmasterol. Also, the enrichment assay indicated that XQLD may act against COVID-19 by controlling immune response, inflammatory response, viral defense, and apoptosis [57].

Stigmasterol, one of the bioactive compounds of *Clinacanthus nutans*, has an immunosuppressive effect on murine cells. Le et al. [104] found that stigmasterol potently suppressed Concanavalin A (ConA)-induced T-cell proliferation and did not block T helper 2 (Th2) and cytokine (IL-4 and IL-10) secretion compared to β-sitosterol. Stigmasterol showed no effect on the secretion of Th1 cytokines (IL-2 and IFN-γ). Thereby, these outcomes provide evidence that phytosterols isolated from *C. nutans* possess immunomodulatory properties with the potential to be used as a novel immunotherapeutic drug [104].

## 7. Neuroprotective Effects

Several research studies have shown that stigmasterol may be a promising bioactive molecule in the protection of neurological disorders (Table 5). As reported in previous investigations, gamma-aminobutyric acid A (GABA_A_) receptors have been involved in neurological disorders. In this sense, Karim et al. [105] investigated the beneficial modulation of stigmasterol isolated from *Artemisia indica* against neurological diseases using in silico docking and other methods. Accordingly, stigmasterol increased GABA-induced currents at the ternary α2β2γ2L, α4β3δ, and binary α4β3 subtypes of GABA_A_R. α4β3δ was found to be notably higher than the binary α4β3 subtype, suggesting that the δ subunit is critical for effectiveness. Moreover, as demonstrated by computational analysis, this sterol exerted a stronger beneficial modulation on the extrasynaptic α4β3δ. In in vivo assays, stigmasterol at 0.5–3.0 mg/kg exhibited potent anticonvulsant and anxiolytic properties in a manner identical to allopregnanolone, thereby demonstrating the implication of a GABAergic mechanism. It seems clear that stigmasterol is considered a steroid drug of interest for the management of neurological diseases due to its ability to positively modulate GABA receptors [105].

Phytosterol is recognized for its ability to penetrate the blood-brain barrier. Using human neuronal cells (SH-SY5Y cells), Pratiwi et al. [61] evaluated in vitro the positive effect of stigmasterol on cell death induced by hydrogen peroxide (H_2_O_2_). There is a significant increase in ROS levels in cells induced by H_2_O_2_ exposure, leading to apoptosis. However, stigmasterol administration preserved ROS levels in cells and inhibited oxidative stress-induced cell death. It was observed that pre-incubation with stigmasterol also promotes the increase in CAT, forkhead box O (FoxO)3a and Bcl-2 in neurons. In a further development, sirtuin 1 (SIRT1) expression levels were also enhanced whereas acetylated lysine levels were reduced, suggesting that SIRT1 function was boosted by stigmasterol. These data stipulate that stigmasterol could conceivably be beneficial in attenuating oxidative stress-induced neurodegeneration [106].

Using the 3-(4,5-dimethylthiazol-2-yl)-2,5-diphenyltetrazolium bromide (MTT) assay, stigmasterol from *Artemisia apiacea* aqueous methanol extract has been shown to exhibit a neuroprotective activity against glutamate-induced neurotoxicity in hippocampal HT-22 cell line through the suppression of calcium ion (Ca^2+^) and ROS generation [61].

On the other hand, Haque et al. [62] investigated whether this constituent might be able to prevent hypoxia/reoxygenation (H/R)-induced excitotoxicity in hippocampal neurons. Consequently, neuronal culture preincubation with stigmasterol (20 μM) was able to provide neuroprotection against excitotoxicity, and alleviated ROS production, DNA damage, and mitochondrial dysfunction. Moreover, stigmasterol treatment also resulted in decreased expression of *N*-methyl-*D*-acetate receptor subunit 2B (GluN2B) and vesicular glutamate transporter 1 (VGLUT1) and decreased size of the recyclable synaptic vesicle (SV) pool. Similar to the ligand-binding domain of the liver X receptor β (LXRβ) agonist GW3695, stigmasterol abolished GluN2B expression. In addition, this phytosterol mediated mitophagy by increasing the expressions of p62, PINK1, and LC3BII. Furthermore, stigmasterol was found to interact with LXRβ via multiple hydrogen bonds, as evidenced by a molecular model study. These results suggest that stigmasterol may have therapeutic potential for ischemic stroke and associated neurological damage.

Animals treated with stigmasterol exhibited a statistically significant reduction in stereotypic behaviors, duration of immobility, locomotor activity, and an observed rise in descent latency. Biochemical assessments showed higher levels of GSH and GABA and lower levels of dopamine, TNF-α, MDA, and acetylcholinesterase (AChE) activity. These results were supported by histopathological alterations in the cortical part of the brain. Furthermore, stigmasterol did not induce catalepsy or adverse effects on the reproductive tract. Thus, it has proven its ability to manage the symptoms of psychosis [59].

Stigmasterol is one of the main agents isolated from *Momordica charantia*. In fact, Pattarachotanant et al. [60] tested the neuroprotective properties of *M. charantia* and evaluated the molecular mechanisms involved in mouse hippocampal neuronal cell line (HT22). The results showed that *M. charantia* exerts neuroprotective activity by reducing ROS generation and decreasing the expression of MAPK proteins cyclin D1, p53, and p38, leading to the suppression of cell apoptosis and the normalization of cell cycle progression. Additionally, stigmasterol showed that its binding to CYP1A1 or CYP1A2 was found to be more effective. The results showed that this plant represents a promising neuroprotective effect, including stigmasterol as a probable active ingredient to avoid neurotoxicity induced by polycyclic aromatic hydrocarbons (PAHs) [60].

Adebiyi’s study [63] aimed to examine the protective activities of ethanol extracts of *Grewia carpinifolia* in vanadium-induced toxicity in mice while noting that β-sitosterol and stigmasterol were the important ingredients isolated from *G. carpinifolia*. The results showed that ethanol extracts of *G. carpinifolia* (200 mg/Kg) enhanced latent time on hanging wire line crossings and decreased rearing in the open field test. Additionally, this extract leads to lower levels of ALT and AST levels, upper levels of PCV, and protection against vanadium-induced disorganization of Purkinje cells in the cerebral cortex. Co-administration of β-sitosterol and stigmasterol markedly alleviated vanadium-induced spatial learning deficits compared with α-tocopherol by decreasing grooming, escape latency, craning, and frequency of stretch and wait for postures. Both compounds enhanced MBP expression and antioxidant enzymes activities such as CAT and SOD as well as decreased oxidative stress markers (MDA, NO, and H_2_O_2_). They also revealed stronger neuroprotective and antioxidant properties and crossed the blood–brain barrier. Consequently, they represent promising compounds for the management of vanadium toxicity [63].

Soares et al. [58] conducted a study to assess the beneficial neuroprotective activity of *Bougainvillea glabra* (Choisy) leaf extract against paraquat (PQ)-induced neurotoxicity. As demonstrated by HPLC and GC–MS methods, *B. glabra* extract showed the presence of several antioxidant compounds including stigmasterol. *B. glabra* extract administration resulted in decreased AChE activity and ROS generation, prevented mortality and dopamine deficiency as well as enhanced locomotor performance and lipid peroxidation. Parkinson’s disease being favored by oxidative stress and/or dopamine depletion. The results obtained by this study could suggest that *B. glabra* extract is an efficient drug in the prevention of neurological disturbances due to the synergistic antioxidant effects of compounds including stigmasterol [58].

On the other hand, Sun et al. [95] analyzed the neuroprotective effect of stigmasterol in an ischemia/reperfusion injury model. Indeed, this molecule was effective in significantly attenuating infarct damage and neurological defects mediated by ischemic/reperfusion injury, ameliorating histopathological alterations, and repairing endogenous antioxidant defense system levels in a dose-dependent manner. At the same time, it promoted mTOR phosphorylation and remarkably inhibited AMPK and JNK phosphorylation, as well as JNK expression and autophagic biomarkers including beclin1 and microtubule-associated protein 1 light chain 3 (LC3) induced by 24 h of reperfusion [95].

In addition, stigmasterol and withanolide-M were identified from *Withania somnifera* screening by molecular docking analysis. Kumar et al. [64] have investigated their eventual neuroprotective properties and their dual inhibitor of inducible/neuronal nitric oxide synthase (iNOS/nNOS). The results reported that this combination showed greater selectivity for iNOS and nNOS compared to their selective inhibitors (AT2 and S19). Both compounds also bound to the catalytic heme domain of iNOS and nNOS with less binding energy (higher affinity). Both compounds exhibit similar binding conformations, as evidenced by AT2 and S19, and form a certain amount of hydrogen bonding and hydrophobic contacts with nNOS and iNOS catalytic sites. Consequently, dual inhibition of iNOS and nNOS without inhibiting eNOS is a potentially beneficial neuroprotective strategy against stroke since nNOS and iNOS are detrimental in neurological disorders, whereas endothelial nitric oxide synthase (eNOS) is beneficial [64].

Stigmasterol from *Tarenna obtusifolia* leaves showed no neuroprotective effect against amyloid-*beta* (Aβ_1–42_)-induced cytotoxicity in neuroblastoma SH-SY5Y cells (13.74% at 50 μM) compared with vomifoliol (55.71% at 50 μM), which revealed neuroprotective activity as demonstrated by the ThT assay. Vomifoliol could therefore provide a novel therapeutic agent to prevent Alzheimer’s disease [107].

Adebiyi et al. [108] isolated bioactive drugs from *Grewia carpinifolia*, and subsequently examined their beneficial effects against vanadium-induced brain damage. The results indicated that stigmasterol, a bioactive ingredient in this plant, significantly promotes improvement in cognitive decline, motor coordination, and improvement in oxidative stress in vanadium-induced neurotoxicity. Stigmasterol significantly (*p* ≤ 0.05) decreased escape latency (28.01 ± 0.02 s) and increased target quadrant swimming time (98.24 ± 17.38 s), respectively, in sodium-metavanadate-induced memory loss in the Morris water maze. It significantly improved exploration and latency in the free field and hanging wire tests, respectively. In addition, the use of stigmasterol stimulated antioxidant enzyme activities, diminished markers of oxidative stress (CAT, SOD, H_2_O_2_) and lipid peroxidation (Malondialdehyde: MDA) in mouse hippocampal homogenates, and increased the expression of myelin basic protein (MBP) [108].

Brimson et al. [65] aimed to explore the protective effect of *Rhinacanthus nasutus* root ethanolic extract against glutamate and amyloid-β25–35 toxicity in cultured mouse clonal hippocampal cells (HT-22). Stigmasterol and other drugs were found to be the bioactive molecules of this extract. They exhibited high free radical scavenging abilities, as well as protected HT-22 cells against glutamate and amyloid-β toxicity. Hence, the synergetic action of various compounds, including stigmasterol, could have antidepressant effects [65].

Since the addition of Amyloid-β (Aβ), a key component of senile plaques in Alzheimer’s disease, stigmasterol has been found to significantly reduce Aβ production in vitro by reducing the expression of all γ-secretase components, by directly decreasing β-secretase activity, reducing cholesterol and presenilin distribution in lipid rafts. On the other hand, positive in vivo effects were reported in the prevention of Alzheimer’s disease when mice were fed a diet high in stigmasterol [66].

## 8. Antibacterial Activity of Stigmasterol

Many experiments promote stigmasterol as a potential antibacterial agent against a broad spectrum of pathogenic strains belonging to Gram-positive and Gram-negative bacteria [21,67,68]. The stigmasterol compound, isolated from the stem bark of *Neocarya macrophylla* from Nigeria, exhibited a potent antibacterial effect described by Yusuf et al. [10] using the agar diffusion and broth micro-dilution methods, in which they recorded an important antibacterial effect of this molecule. At a concentration of 100 µg/mL, this compound was able to inhibit the growth of all organisms tested (Φ = 23–30 mm), namely *S. aureus*, *Streptococcus faecalis*, *Escherichia coli*, and *Pseudomonas fluorescens*, except vancomycin-resistant *enterococci*, *Salmonella typhi*, and *Klebsiella pneumonia*. The minimum inhibitory concentration (MIC) values were low (6.25–25 µg/mL), concomitantly with the *minimum bactericidal concentration* (MBC) which ranges from 12.5 to 50 µg/mL, indicating that the compound has very good antibacterial activity against the pathogenic organisms [17]. In addition, *Staphylococcus aureus*, *Staphylococcus aureus*, *Streptococcus pyrogenes*, *Bacillus subtilis*, *Corynebacterium ulcerans*, *Escherichia coli*, *Proteus mirabilis*, *Proteus vulgaris*, *Pseudomonas aeruginosa*, *Salmonella typhi*, and *Shigellia dysenteriae* were inhibited at modest concentrations of stigmasterol (50 µg/mL), derived from *Spillanthes acmella*, in which the MIC was 12.5 µg/mL with diameters of 20 ± 0.1 and 24 ±0.1 mm [71]. In another experiment, Abdissa et al. [109] showed that stigmasterol obtained from *Caylusea abyssinica* roots was able to repress *Staphylococcus aureus* (ATCC25903), *Escherichia coli* (ATCC25722), *Pseudomonas aeruginosa* (DSMZ1117), and *Salmonella thyphimurium* (ATCC13311)) with inhibition diameters between 11 and 18 mm [110]. In another study, the antibacterial activity of this substance was evaluated on three pathogenic bacteria: *Staphylococcus aureus* ATCC25922, *Enterococcus faecalis* ATCC10541, and *Salmonella typhi* ATCC6539. The researchers demonstrated that stigmasterol is effective against all strains tested with MIC values between 6.25 and 1000 µg/mL [109]. Moreover, Awouafack et al. [111] examined the bacteriostatic activity of stigmasterol extracted from the crude ethanol extract of *Eriosema robustum* twigs against four bacterial cultures and found that stigmasterol exhibits strong bacteriostatic activity on *Pseudomonas aeruginosa* (60 µg/mL), and a moderate action on *Staphylococcus aureus* (250 µg/mL), *Escherichia coli* and *Enterococcus faecalis* (250 µg/mL). Interestingly, a study reported that steroids are able to inhibit bacterial cell surface protein, thus preventing transpeptidation [69]. More specifically, stigmasterol from the plant *Phyllanthus columnaris* has been shown to exhibit bacteriostatic properties against MRSA through a reduction in the translation processes leading to the inhibition of protein synthesis and prevention of bacterial growth [67].

## 9. Antifungal Activity

Various studies have reported that botanicals rich in stigmasterol possess antifungal properties [70,71]. An important antifungal impact was measured on two fungi strains; *Candida albicans* and *Candida krusei* where the stigmasterol inhibition zone diameters were 25 and 27 mm, and the MIC values were 12.5 and 25 µg/mL, respectively [17]. These outcomes were in line with other research, where they found that stigmasterol was able to inhibit *Candida albicans* and two other *Candida* species (*C. virusei* and *C. tropicalis*) with minimum inhibitory and fungicidal concentrations of 12.5 µg/mL and 50 µg/mL, respectively [71]. Conversely, other findings stated that this phytosterol extracted from the root bark of *Zanthoxylum paracanthum* does not inhibit the growth of *C. albicans* (MIC > 1000 μg/mL) [112]. In addition, it revealed a great inhibitory activity on three pathogenic fungal species, namely, *Aspergillus flavus*, *Penicillium digitatum*, and *Fusarium verticilloides* with inhibition diameters ranging from 14 to 18 mm [72].

## 10. Antioxidant Activity

Stigmasterol has shown antioxidant properties in numerous investigations (in vivo and in vitro). One of the first researches on the antioxidant properties of stigmasterol was carried out by Hassanein et al. [113] on *Vicia faba* previously exposed to salt stress. Findings revealed that stigmasterol treatment (seeds pre-treated with 500 µM of stigmasterol) reduced oxidative damage by increasing the enzymatic antioxidant system (CAT and ascorbate peroxidase (APX)) and the non-enzymatic antioxidant system (GSH). Whereas, in control plants subjected to stressful conditions, the defense mechanisms were not sufficient to stabilize oxidative damage. Furthermore, Osuntokun et al. [114] showed that stigmasterol isolated from ethyl acetate stem bark extract of *Spondias mombin* acts synergistically with a phytosterol to reduce ROS production in diabetic rats and some oxidoreductase enzymes, including CAT, glutathione reductase (GR), glutathione peroxidase (GPX), and 6-phosphate dehydrogenase, which are considered an important cause of cellular oxidative stress. The same work revealed that stigmasterol can bind to redox sensor complex and Kelch-like ECH Associated Protein 1 (KEAP 1) *that* act as a key sensor of oxidative and electrophilic stress in Wistar rat liver cells, to reduce the oxidative stress of electrophiles endoplasmic reticulum stress from ROS. More interestingly, Liang et al. [95,115] studied the effect of stigmasterol on a reperfusion model of middle cerebral artery occlusion in rats where the free radical production in brain tissue was increased. The outcomes of this work indicated that stigmasterol can noticeably prevent the decrease in SOD, CAT, and glutathione-peroxydase (GSH-Px) and the increase in *malondialdehyde* (MDA) in serum and brain tissue of model rats after cerebral ischemia and reperfusion in a dose-dependent manner (20–80 mg/kg) [95,115]. Moreover, some free radical scavenging assays have clearly revealed that the stigmasterol of *Clerodendron inerme* chloroform extract was able to inhibit the DPPH radical with an IC_50_ of 220 µg/mL. This compound also showed potent superoxide radical and hydroxyl radical scavenging activities with IC_50_ values of 210 and 150 µg/mL, respectively. While standard quercetin was more potent than stigmasterol, it exhibited IC_50_ values ranging from 110 to 135 µg/mL [116].

## 11. Anti-Diabetic Effects

Stigmasterol has also been found to exert anti-diabetic effects. Indeed, Wang et al. [117] showed that stigmasterol extracted from soybean oil can significantly improve GLUT4 translocation and glucose uptake in L6 cells, after supplementation of 50 and 100 mg/kg of this substance to diabetic KK-Ay mice for 4 weeks, indicating that the hyperglycemic phenotype of diabetic mice significantly improved after stigmasterol treatment. Moreover, the same experiment showed a notable hypoglycemic effect by the reduction in fasting blood glucose, serum insulin levels, and oral glucose tolerance in the treated groups. Another study reported that diabetic rats supplemented with 0.25 and 0.50 mg/kg of stigmasterol for 21 days, showed a considerable decrease in fasting blood glucose levels associated with an increase in serum insulin [30]. Moreover, Ramu et al. [118] showed that stigmasterol reduced fasting blood glucose levels and moderated abnormalities in serum/urine protein, urea and creatinine, in an alloxan-induced diabetic rat model after treatment for 4 weeks with 100 and 200 mg/kg stigmasterol solution. Also, some diabetic symptoms such as polyphagia, polyuria, urinary glucose, and body weight loss were ameliorated in the diabetic rat group. Insulin and hemoglobin levels were also shown to be increased, whereas the HbA1c level was reduced. Furthermore, the altered activities of gluconeogenic enzymes (glucose-6-phosphatase, fructose-1,6-bisphosphatase and lactate dehydrogenase) significantly returned to normal levels. Moreover, histological observations revealed marked regeneration of β-cells in drug-treated diabetic rats. Panda et al. [119] conducted their research to examine the effect of stigmasterol on diabetic female mice. Oral treatment with stigmasterol (2.6 mg/kg b.w.) for 20 days reduced serum glucose concentrations as well as hepatic glucose-6-phophatase (G-6-Pase) activity with a concomitant increase in insulin level. Another study showed that stigmasterol also increases total insulin and improves insulin secretion in cells exposed to glucolipotoxicity [120]. Recently, Reza et al. [121] reported the possible mechanism underlying the anti-diabetic properties of stigmasterol-rich *Aeginetia indica*. The molecular docking analyses revealed stigmasterol binding to sirtuin 4, an NAD-dependent deacylase enzyme that down-regulates leucine- and glutamate-dehydrogenase-induced insulin secretion.

## 12. Antiparasitic Activity

The structural features of stigmasterol appear to be crucial against some parasite strains. The in vivo effects of stigmasterol against *Trypanosoma congolense* in addition to its trypanosomal sialidase inhibitory effects were evaluated. Stigmasterol (200 mg/kg) has been found to significantly reduce parasitemia and improve *T. congolense* induced anemia as well as liver and kidney damage. Furthermore, the treatment with stigmasterol prevented the *T. congolense*-associated increase in free serum sialic acid with a corresponding decrease in membrane-bound sialic acid. Subsequently, in vitro enzyme kinetic studies revealed that stigmasterol is an uncompetitive inhibitor of a partially purified bloodstream *T. congolense* sialidase with an inhibition binding constant of 356.59 mM. Moreover, stigmasterol formed a single hydrogen bond interaction with a major residue (D63) at the catalytic domain of *T. rangeli* sialidase, showing that stigmasterol could retard the proliferation and the major pathological features of *T. congolense* infection [73]. Another study assessed the in vitro inhibitory effects of stigmasterol on promastigotes and amastigotes of *Leishmania major* (MHOM/IR/75/ER) using the MTT assay. Stigmasterol had efficient adverse effects on promastigotes and amastigotes of *L. major* with a fatality rate of 71.96% at 24 h post-culture in a concentration of 50 μg/mL [74].

## 13. Antiviral Activity of Stigmasterol

Stigmasterol has been shown to have antiviral properties. Indeed, Soekamto et al. [122] investigated the antiviral effect of this compound isolated from the stem bark extract of *Melochia umbellate* against dengue virus (DENV-2). The Dengue Antivirus Test revealed a potent inhibitory activity of this compound (IC_50_ = 9.11 μg/mL). Other studies explored the antiviral properties of stigmasterol derivatives. Specifically, Petrera et al. [123] tested two stigmasterol derivatives, (22S,23S)-22,23-dihydroxystigmast-4-en-3-one and (22S,23S)-3βbromo-5α,22,23-trihydroxystigmastan-6-one against herpes simplex virus type1 (HSV-1). Both compounds inhibited HSV-1 replication and spreading in human epithelial cells derived from ocular tissues. Another study examined the effect of 7-ketostigmasterol isolated from the green Antarctic alga *Prasiola crispa* on the replication of equine herpesvirus 1 (EHV-1). Data showed that stigmasterol inhibited EHV-1 in a dose-dependent manner. The inactivation percentages were 12.5 (51%), 25 (67%), 50 (91%), and 100 μM (100%). It could be deduced that 7-keto-stigmasterol probably interferes with EHV-1 attachment to cells with irreversible inactivation of virus infectivity [124].

## 14. Bioaccessibility and Bioavailability

It has been established that the level of phytosterols in natural foods is not very high, and phytosterols are not soluble in water. The solubility of phytosterols in dietary oil is only about 1% [125]. To deal with this issue, several researchers have attempted to identify alternatives to improve the bioavailability of phytosterols, including stigmasterol. Indeed, the esterification process was an efficient tool to increase the solubility of phytosterols in oil. This is the case of phytosterol oleate ester (PO), phytosterol acetic ester (PA) and phytosterol linoleic ester (PL), and other esters of phytosterol, which have been synthetically produced from phytosterol (PS) and have enhance the total content of phytosterol in food and the solubility in oil [126]. In humans, phytosterols (in esterified or non-esterified form) are principally transported in the blood by LDL, in contrast to rats, where HDL is the major transporter. Unabsorbed phytosterols are excreted mainly unchanged in the feces [127]. It is important to signal that phytosterol esters can be converted in the human body to phytosterols and fatty acids after digestion, and have unchanged biological functions. After entering the human body, phytosterol esters are emulsified by bile and hydrolyzed by lipase. Subsequently, phytosterol esters are absorbed into the bloodstream and transported to the tissues by intestinal absorption. They are then assimilated and utilized within the human body to perform their pharmacological and biological effects [128]. In this context, Wang et al. in their recent study aimed to investigate the bioavailability of synthesized phytosterol esters (stigmasterol as the main constituent) in vitro and in vivo using Caco-2 cell model and Wistar rats. The results showed that the bioavailability of phytosterol after esterification was better in vivo and in vitro, especially for the linoleic ester of phytosterol, which had a bioavailability of four times higher than that of free phytosterol [129]. Considering that phytosterols represent plant sterols structurally similar cholesterol, they can affect the bioaccessibility and bioavailability of cholesterol. Therefore, investigations into the distribution, metabolism, and excretion properties of phytosterols include quantifying the disponibility of cholesterol and comparing the bioavailability of phytosterols with that of cholesterol. In this sense [130], investigated the impact of increased bile salts and the addition of key lipid metabolism enzymes such as gastric lipase and cholesterol esterase on the bioaccessibility of sterols, including stigmasterol, in a plant-sterol-enriched beverage using an in vitro digestion scenario (INFOGEST). The results showed that the total bioaccessibility values of plant sterols obtained from the digestion of the plant sterol-enriched beverage with gastric lipase and cholesterol esterase (0.075 U/mL intestinal digesta) (8.0%) are closer to the absorption in humans (6%), as shown in the study by Kritchevsky and Chen [131]. In addition, the bioaccessibility values of stigmasterol obtained (5.5%) were similar to the absorption rates of stigmasterol (4.8%) reported in human studies [132]. In male rats with thoracic duct cannulation and cholesterol and phytosterol emulsions, only 0.5% of stigmasterol was recovered in the lymph, compared with cholesterol, campesterol, brassicasterol, sitosterol, and sitostanol, for which the percentages obtained were 59.2, 15.4, 3.3, 2.4, and 0.3%, respectively [133]. Overall, studies in both rats and humans indicate that the bioavailability of phytosterols and is influenced by the form of administration [127].

## 15. Toxicology and Clinical Investigations

The Panel on Food Additives and Nutrient Sources added to Food (ANS) observed that phytosterols are used as emerging new dietary components to lower high serum LDL cholesterol levels, which leads to already fairly high daily intakes of up to about 3 g/day. Furthermore, according to the panel, the estimated average intake of stigmasterol-rich plant sterols is 0.6–11.4 mg/day (0.01–0.2 mg/kg bw/day) for adults and 0.6–8.4 mg/day (0.01–0.1 mg/kg bw/day) for the elderly. The ranges of high-percentile (95%) intakes are 24–443.3 mg/day (0.4–7.4 mg/kg bw/day) for adults and 28.2–192.6 mg/day (0.5–3.2 mg/kg bw/day) for the elderly [134]. In a European investigation, Sioen et al. estimated regional intakes of plant sterol-enriched foods and supplements containing plant sterols that are commonly distributed in Belgium and reported a mean intake of 0.70 g/day for preschoolers and 1.51 g/day for adults. The 95th percentile intake for adults was considered to be 4.2 g/day, and 16.4% of the adult population had a plant sterol intake greater than 3 g/day [135].

With the aim to assess the toxicological effects of free phytostreols and their esters in vivo. In a subchronic toxicity study, Alpk:APfSD (Wistar-derived) rats (20/sex/group) were fed diets containing phytosterol esters at levels of 0, 0.16, 1.6, 3.2 and 8.1% (*w*/*w*) in the diet (equivalent to phytosterol concentrations of 0, 0.1, 1, 2 or 5%, respectively) for 90 days. The phytosterols used are derived from a variety of common edible vegetable oil distillates (mainly soya bean) and re-esterified with fatty acids from sunflower oil to increase their oil solubility. The phytosterol profile in the mixture was mainly β-sitosterol (48.7%), stigmasterol (21.6%), and campesterol (25.8%), with minor amounts of β-sitostanol (1.4%), brassicasterol (1.1%), unknowns (0.8%), and cholesterol (0.4%). The consumption of phytosterol esters, phytosterols, and stigmasterol were evaluated. During the study, there were no effects on mortality, nor clinical signs of toxicity, food consumption or utilization, body weights, or organ weights that were attributed to the consumption of phytosterol esters. From the content of stigmasterol in the phytosterol ester mixture (21.6%), the authors calculated a no-observed-adverse-effect level (NOAEL) expressed as stigmasterol equivalent to approximately 879 mg/kg bw/day, the highest dose tested. Minor statistically significant changes were observed in some hematological and clinical chemistry parameters. The authors considered these changes to be of no toxicological significance [136].

Another study had the objective to investigate in vivo the potential subchronic toxicity of plant sterol esters following daily oral gavage administration to male and female Sprague Dawley rats (10 to 16/sex/group) at dose levels of 0, 1000, 3000, and 9000 mg phytosterol esters/kg bw/day for 90 days. The plant sterols were isolated from soybeans and subsequently esterified with unsaturated fatty acids (oleic acid ≥ 70%) from olive oil to increase their oil solubility. The plant sterol esters were composed of 49.4% β-sitosterol, 27.9% campesterol, and 18.5% stigmasterol as well as other agents. At the end of the experiment, the authors concluded that there were no adverse effects on mortality, clinical signs of toxicity, food and water consumption, ophthalmoscopy, urinalysis, haematology, serum biochemistry, necropsy findings, and organ weights in any treatment group. Due to the suppression of body weight gains in both sexes and the observed infiltration of mononuclear cells in the heart in males at a dose level of 9000 mg phytosterol esters/kg bw/day, the NOAEL for both sexes was considered to be 3000 mg phytosterol esters/kg bw/day [137].

Globally, many investigations report no important side effects of phytosterols on human including stigmasterol. Nevertheless, toxicity studies on stigmasterol-rich plant sterols still missing, and investigations of phytosterols and phytosterol esters to date have been limited to 90-day subchronic toxicity tests in rats, as evidenced in the data obtained in some studies [127,136].

## 16. Conclusions and Perspectives

Here, we reported biological effects and pharmacological properties of stigmasterol. This compound showed stochastic versatility of biological effects by exhibiting numerous health benefits. The involved mechanisms of stigmasterol are multiple actions including sub-cellular, cellular, and molecular targets. Indeed, this bioactive compound inhibited several human cancer cell lines in vitro and in vivo via its different mechanisms, including apoptosis, cell cycle arrest, and autophagy. However, anticancer effects of stigmasterol should be completed by other investigations, as well the determination of pharmacodynamic actions and pharmacokinetic ways. On the other hand, in vivo studies of stigmasterol in other pharmacological systems, such as anti-inflammatory and antidiabetic systems, should be carried to investigate more importantly the effect of this compound. In addition, clinical investigations should be also explored for further applications of stigmasterol in pharmaceutical applications. Moreover, toxicological explorations using in vivo models, as well as human patients, will be examined to validate the safety of this natural drug.

## Figures and Tables

**Table 3:** Anti-inflammatory effects of stigmasterol.

Experimental Approaches	Key Results	References
Collagen-induced arthritis (CIA) induced inflammation in rats	Significantly suppressed TNF-α/IL-6/IL-1β/Inos/COX-2 expression Increased the expression of anti-inflammatory cytokine IL-10 Down-regulated the expression of NF-kBp65/p38MAPK Inhibited p-IKB-α activation	[101]
IL-1β-induced inflammation in rat chondrocytes	Decreased the expression of iNOS and IL-6	[46]
Dimethylbenzene-induced ear edema test	Inhibition = 50.34% at 30 mg/kg dose Inhibited ear edema Showed significant anti-inflammatory activity (*p* < 0.01)	[48]
Carrageenan-induced peritonitis and arachidonic acid-induced paw edema Molecular docking Acetic acid-induced writhing test	Decreased leukocyte infiltration in peritonitis assay Reduced paw edema elicited by arachidonic acid Prevented abdominal writhes and paw licking Reduced the number of crossings Presented anti-inflammatory effects mediated by glucocorticoid receptors	[51]
Dermatitis induced by 12-O-tetradecanoylphorbol-13-acetate (TPA)	Controlled inflammatory features such as ear skin oedema and neutrophilia Inhibited the expression of pro-inflammatory cytokines Reduced serum levels of TNF-α	[49]
Induced rat paw oedema model	Inhibition = 79.86 ± 0.16% Inhibited the production of pro-inflammatory proteins Exhibited the best binding energy (6.5 kcal/mol) against TNF-α Observed to be an effective molecule against the COX-2 enzyme Used as a topical anti-inflammatory drug Enhanced electrophilic index	[52]
Ovalbumin induced allergic asthmatic mice	Improved the number of inflammatory Decreased the level of inflammatory modulator TNF-α and IgE antibodies Reduced IL- 4, IL-5, and IL-13 Increased the expression levels of AQP1 and AQP5	[55]
LPS-stimulated RAW264.7 cells LPS-induced acute lung injury in C57BL/6J mice	Activated the LXRs/ABCA1 signaling pathway Affected the activation of the TLR4/NF-κB pathway	[102]
Carrageenan-induced hind paw edema model	Displayed anti-inflammatory effect (*p* < 0.001) Decreased NO production Inhibited TNF-α, IL-1β, IL-6, and COX-2 production	[54].

**Table 4:** Immunomodulatory effects of stigmasterol.

Experimental Approaches	Key Results	References
Cyclophosphamide-induced immunosuppression in mice	Exhibited effective immunomodulatory effect Potentiated non-specific immune response Increased haematological parameter Increased neutrophil adhesion test	[26]
Ovalbumin-induced airway	Decreased the proliferation of lymphocytes, eosinophils, and monocytes Decreased the perivascular, peribronchiolar, and alveolar infiltration of inflammatory cells Reduced the expression of VCAM-1/OVA sIgE levels	[103]
Dextran sodium sulfate (DSS)-induced colitis model	Reduced DSS-induced inflammatory responses Regulated the Treg/Th17 balance Up-regulated IL-10 and TGF-β expression Decreased the expression of IL-17A Suppressed the Th17 cell response Promoted the development of Treg cells Provoked higher generation of gut microbiota-derived short-chain fatty acids (SCFAs), specifically butyrate Activated PPARγ by butyrate restored Treg/Th17 cells balance	[53]
Carrageenan-induced hind paw edema model in NIH mice	Reduced macrophage release of TNF-α/NO/COX-2/IL-1β/IL-6 Did not alter macrophage viability	[54]
Network pharmacology data	Targeted PI3K-Akt signaling pathway, the TNF-α and IL-17 signaling pathway, and Th17 cell differentiation Protected against myasthenia gravis	[56]
Biological network analysis	Targeted STAT3, CCL2, MAPK1, FOS, VEGFA, CASP3, IL-6, MAPK3, MAPK8, and CASP8 Acted against COVID-19 Controlled immune, inflammatory response and apoptosis	[57]
Murine cells	Suppressed Concanavalin A (ConA)-induced T-cell proliferation Did not block T helper 2 (Th2) and cytokine (IL-4 and IL-10) secretion Showed no effect on the secretion of Th1 cytokines (IL-2 and IFN-γ)	[104]

**Table 5:** Neuroprotective effects of stigmasterol.

Experimental Approaches	Key Results	References
In silico docking Recombinant GABA_A_ receptor subtypes expressed in Xenopus laevis oocytes	Up-regulated GABA-induced currents at the ternary α2β2γ2L, α4β3δ, and binary α4β3 subtypes of GABA_A_R	[105]
Human neuronal cells (SH-SY5Y cells)	Maintained ROS levels inside the cells Prevented oxidative stress-induced cell death Facilitated the up-regulation of FoxO3a/CAT/Bcl-2 in the neurons Increased the expression levels of sirtuin 1 (SIRT1) Decreased acetylated lysine levels Stimulated SIRT1 activity	[61]
Glutamate-induced neurotoxicity in hippocampal HT-22 cell line	Exhibited neuroprotective activity Inhibited ROS production Inhibited Ca^2+^ production	[106]
Hypoxia/reoxygenation (H/R)-induced excitotoxicity in hippocampal neurons	Protected against excitotoxicity Attenuated oxidative stress Attenuated mitochondrial dysfunction and DNA damage Controlled vesicle exocytosis Reduced VGLUT1 expression and vesicle pool size Suppressed GluN2B expression Up-regulated mitophagy-related proteins: LC3BII/p62/PINK1	[62]
Ketamine-induced psychotic symptoms in mice	Decreased locomotor activity Reduced stereotypic behaviors Decreased immobility duration Increased step-down latency Increased GABA and GSH levels Decreased dopamine/MDA/TNF-α/AChE activity Did not found to cause catalepsy	[59]
Hippocampal neuronal cell line (HT22) In silico analysis	Reduced ROS production Down-regulated cyclin D1/p53/p38/MAPK protein expressions Inhibited cell apoptosis Normalized cell cycle progression Inhibited cytochrome P450 (CYP: CYP1A1, CYP1A2, and CYP1B1) Revealed high binding to CYP1A1/CYP1A2	[60]
Vanadium-induced toxicity in mice	Attenuated spatial learning deficits Reduced escape latency Reduced grooming and rearing Reduced stretch-attend posture frequency Increased activities of CAT/SOD Decreased oxidative stress markers Increased MBP expression Crossed the blood-brain barrier Exhibited potent antioxidant Exhibited neuroprotective activities	[63]
Paraquat exposure-induced Parkinson’s disease-like symptoms and oxidative stress in Drosophila melanogaster	Decreased AChE activity and ROS generation Prevented mortality and dopamine deficiency Enhanced locomotor performance and lipid peroxidation	[58]
Induced Cerebral Ischemic/Reperfusion Injury in rats	Reduced neurological deficits and infarct damage Improved histopathology changes Restored the levels of the endogenous antioxidant defense system Depressed the expression level of beclin1, and the conversion of LC3 I to LC3 II Promoted the phosphorylation of mTOR Inhibited the phosphorylation of AMPK/JNK and expression of JNK	[95]
Structure-based molecular docking	Showed higher selectivity for iNOS and nNOS Exhibited similar binding conformations Inhibited iNOS and nNOS without inhibiting eNOS	[64]
Neuroblastoma SH-SY5Y cells	Inhibition % = 13.74% at 50 μM Did not exhibit neuroprotective potential in Aβ1–42-treated SH-SY5Y cells	[107]
Vanadium-induced neurotoxicity	Decreased escape latency 28.01 ± 0.02 Increased swimming time in target quadrant 98.24 ± 17.38 Increased exploration and latency Increased activities of antioxidant enzymes Decreased oxidative stress markers and lipid peroxidation Increased MBP expression	[108]
HT-22 mouse hippocampal cells	Attenuated the neuron cell death Protected against glutamate toxicity	[65]
-	Reduced Aβ generation Decreased β-secretase activity Reduced expression of all γ-secretase components Reduced cholesterol and presenilin distribution in lipid rafts Decreased BACE1 internalization to endosomal compartments	[66]
